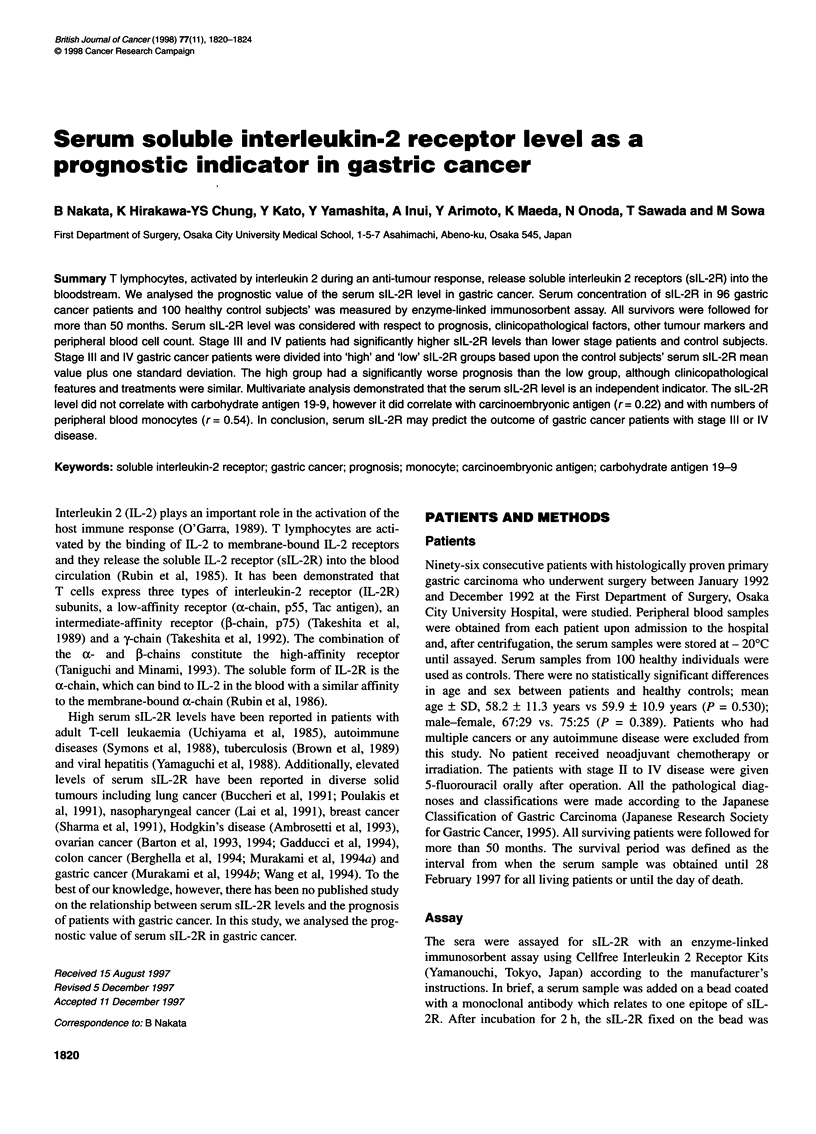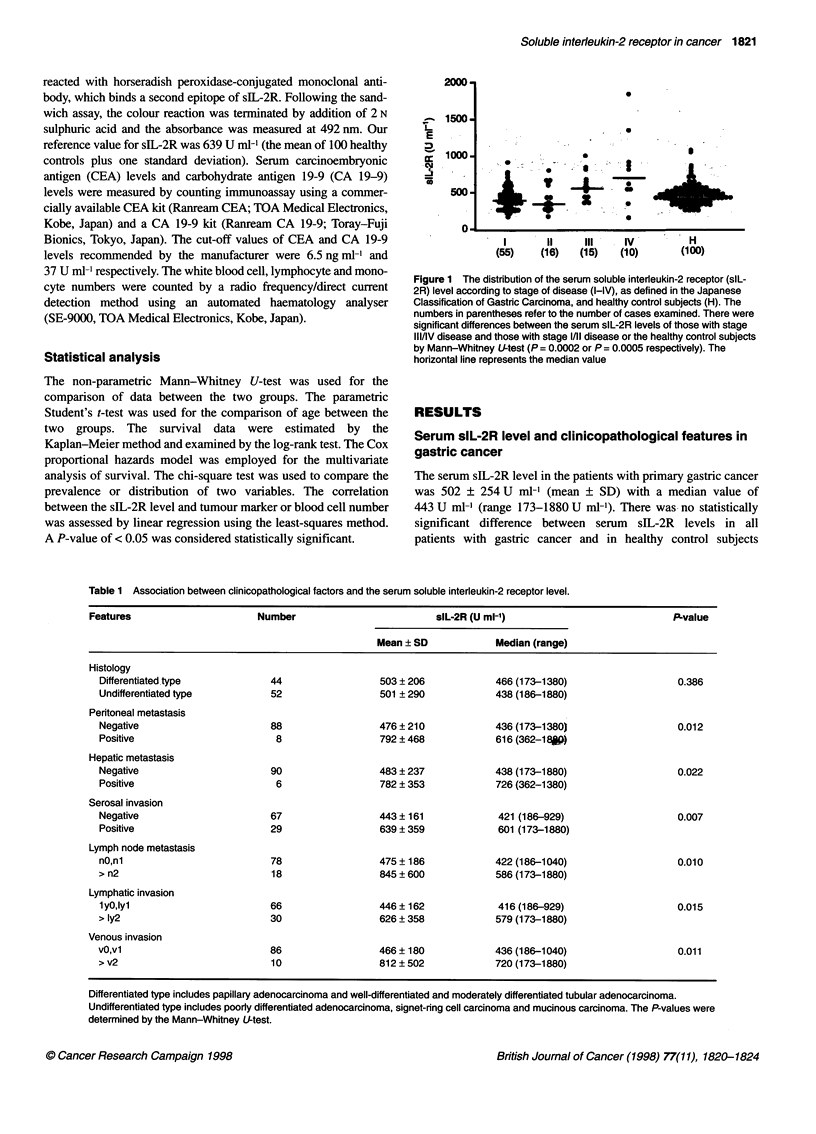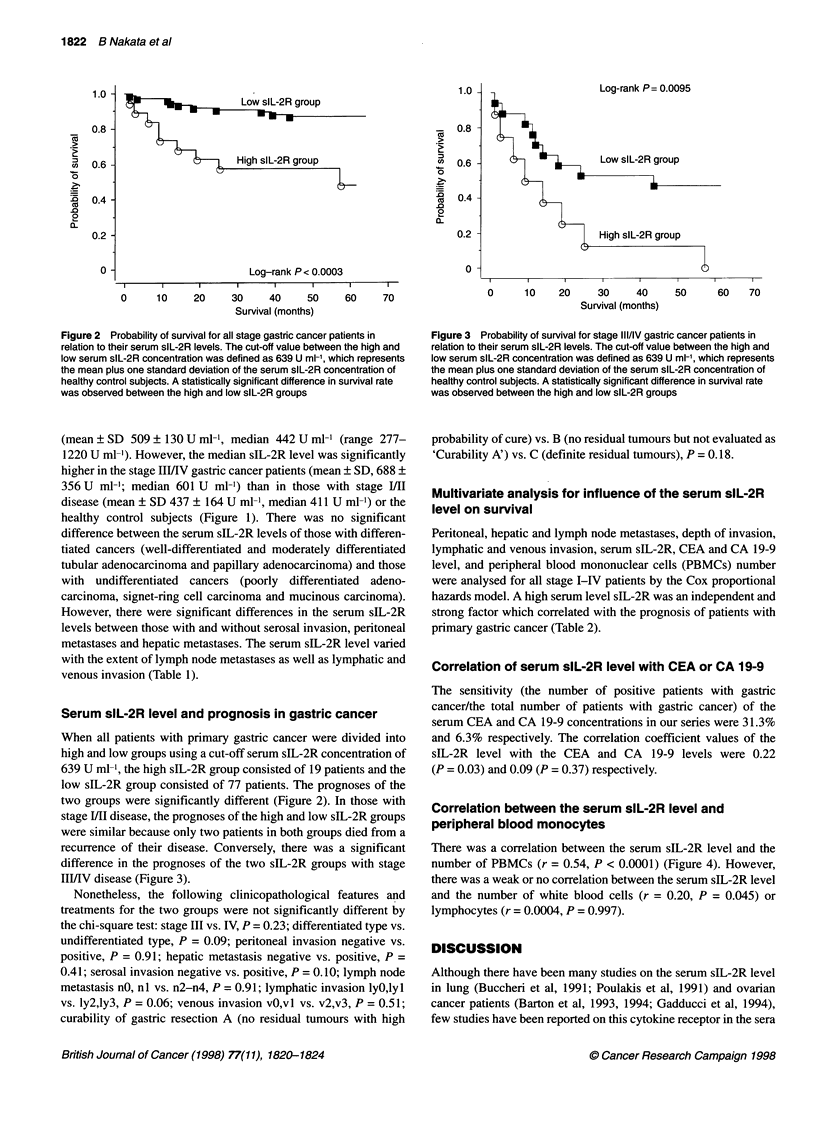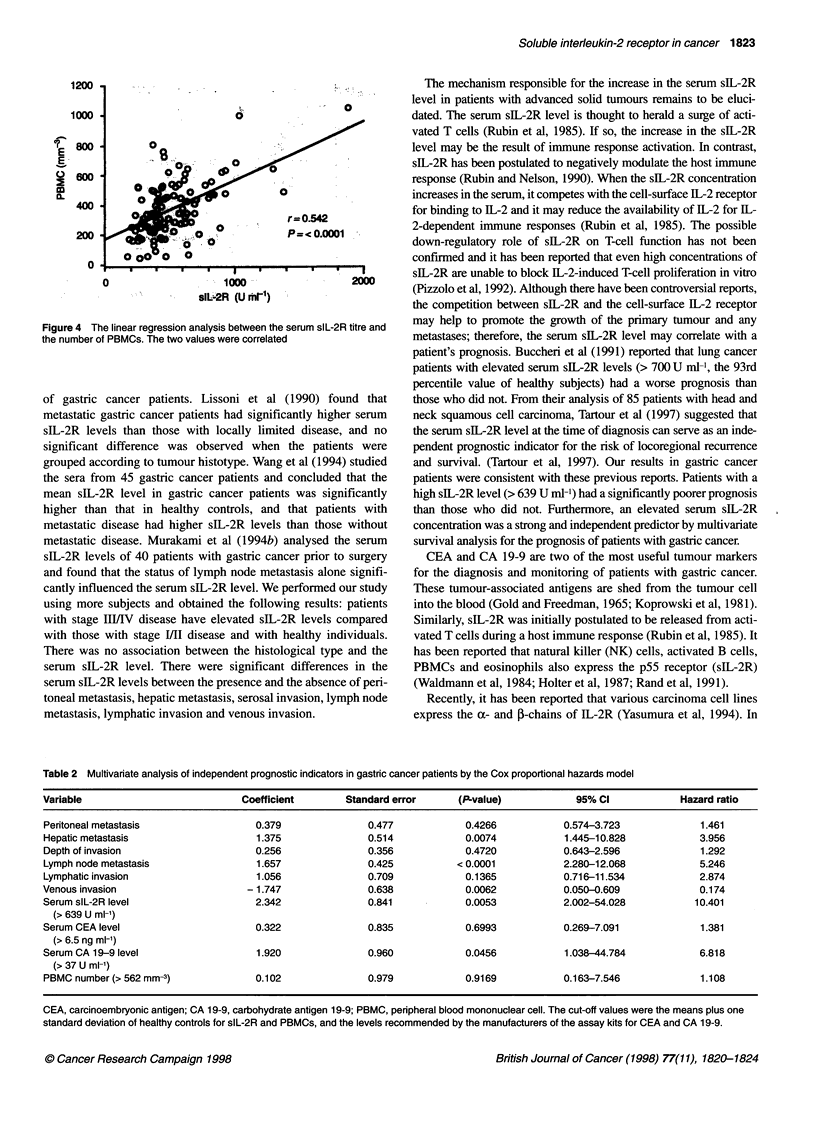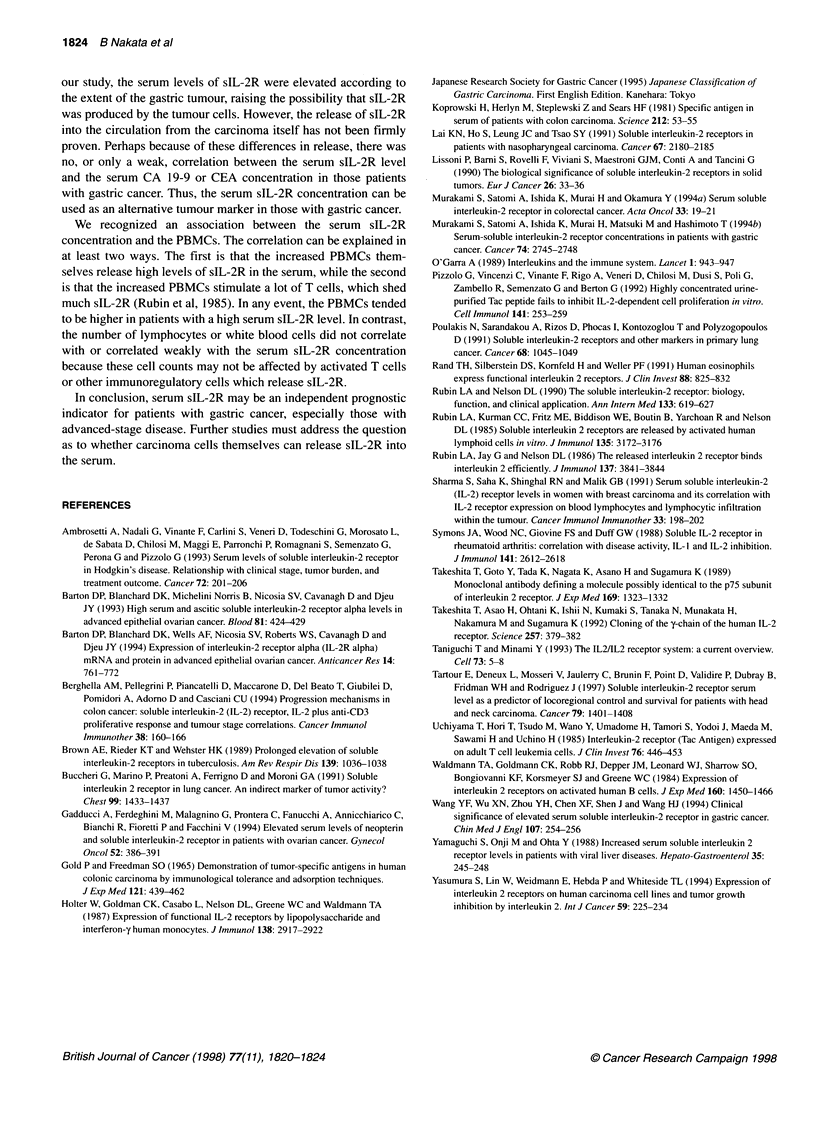# Serum soluble interleukin-2 receptor level as a prognostic indicator in gastric cancer.

**DOI:** 10.1038/bjc.1998.302

**Published:** 1998-06

**Authors:** B. Nakata, K. H. Chung, Y. Kato, Y. Yamashita, A. Inui, Y. Arimoto, K. Maeda, N. Onoda, T. Sawada, M. Sowa

**Affiliations:** First Department of Surgery, Osaka City University Medical School, Osaka, Japan.

## Abstract

T lymphocytes, activated by interleukin 2 during an anti-tumour response, release soluble interleukin 2 receptors (sIL-2R) into the bloodstream. We analysed the prognostic value of the serum sIL-2R level in gastric cancer. Serum concentration of sIL-2R in 96 gastric cancer patients and 100 healthy control subjects' was measured by enzyme-linked immunosorbent assay. All survivors were followed for more than 50 months. Serum sIL-2R level was considered with respect to prognosis, clinicopathological factors, other tumour markers and peripheral blood cell count. Stage III and IV patients had significantly higher sIL-2R levels than lower stage patients and control subjects. Stage III and IV gastric cancer patients were divided into 'high' and 'low' slL-2R groups based upon the control subjects' serum sIL-2R mean value plus one standard deviation. The high group had a significantly worse prognosis than the low group, although clinicopathological features and treatments were similar. Multivariate analysis demonstrated that the serum sIL-2R level is an independent indicator. The sIL-2R level did not correlate with carbohydrate antigen 19-9, however it did correlate with carcinoembryonic antigen (r = 0.22) and with numbers of peripheral blood monocytes (r = 0.54). In conclusion, serum sIL-2R may predict the outcome of gastric cancer patients with stage III or IV disease.


British Journal of Cancer (1998) 77(11), 1820–1824
© 1998 Cancer Research Campaign

### Serum soluble interleukin-2 receptor level as a prognostic indicator in gastric cancer

**B Nakata, K Hirakawa-YS Chung, Y Kato, Y Yamashita, A Inui, Y Arimoto, K Maeda, N Onoda, T Sawada and M Sowa**

First Department of Surgery, Osaka City University Medical School, 1-5-7 Asahimachi, Abeno-ku, Osaka 545, Japan

**Summary** T lymphocytes, activated by interleukin 2 during an anti-tumour response, release soluble interleukin 2 receptors (sIL-2R) into the bloodstream. We analysed the prognostic value of the serum sIL-2R level in gastric cancer. Serum concentration of sIL-2R in 96 gastric cancer patients and 100 healthy control subjects' was measured by enzyme-linked immunosorbent assay. All survivors were followed for more than 50 months. Serum sIL-2R level was considered with respect to prognosis, clinicopathological factors, other tumour markers and peripheral blood cell count. Stage III and IV patients had significantly higher sIL-2R levels than lower stage patients and control subjects. Stage III and IV gastric cancer patients were divided into 'high' and 'low' sIL-2R groups based upon the control subjects' serum sIL-2R mean value plus one standard deviation. The high group had a significantly worse prognosis than the low group, although clinicopathological features and treatments were similar. Multivariate analysis demonstrated that the serum sIL-2R level is an independent indicator. The sIL-2R level did not correlate with carbohydrate antigen 19-9, however it did correlate with carcinoembryonic antigen ($r = 0.22$) and with numbers of peripheral blood monocytes ($r = 0.54$). In conclusion, serum sIL-2R may predict the outcome of gastric cancer patients with stage III or IV disease.

**Keywords:** soluble interleukin-2 receptor; gastric cancer; prognosis; monocyte; carcinoembryonic antigen; carbohydrate antigen 19–9

Interleukin 2 (IL-2) plays an important role in the activation of the host immune response (O'Garra, 1989). T lymphocytes are activated by the binding of IL-2 to membrane-bound IL-2 receptors and they release the soluble IL-2 receptor (sIL-2R) into the blood circulation (Rubin et al, 1985). It has been demonstrated that T cells express three types of interleukin-2 receptor (IL-2R) subunits, a low-affinity receptor (α-chain, p55, Tac antigen), an intermediate-affinity receptor (β-chain, p75) (Takeshita et al, 1989) and a γ-chain (Takeshita et al, 1992). The combination of the α- and β-chains constitute the high-affinity receptor (Taniguchi and Minami, 1993). The soluble form of IL-2R is the α-chain, which can bind to IL-2 in the blood with a similar affinity to the membrane-bound α-chain (Rubin et al, 1986).

High serum sIL-2R levels have been reported in patients with adult T-cell leukaemia (Uchiyama et al, 1985), autoimmune diseases (Symons et al, 1988), tuberculosis (Brown et al, 1989) and viral hepatitis (Yamaguchi et al, 1988). Additionally, elevated levels of serum sIL-2R have been reported in diverse solid tumours including lung cancer (Buccheri et al, 1991; Poulakis et al, 1991), nasopharyngeal cancer (Lai et al, 1991), breast cancer (Sharma et al, 1991), Hodgkin's disease (Ambrosetti et al, 1993), ovarian cancer (Barton et al, 1993, 1994; Gadducci et al, 1994), colon cancer (Berghella et al, 1994; Murakami et al, 1994*a*) and gastric cancer (Murakami et al, 1994*b*; Wang et al, 1994). To the best of our knowledge, however, there has been no published study on the relationship between serum sIL-2R levels and the prognosis of patients with gastric cancer. In this study, we analysed the prognostic value of serum sIL-2R in gastric cancer.

*Received 15 August 1997*
*Revised 5 December 1997*
*Accepted 11 December 1997*

*Correspondence to:* B Nakata

### PATIENTS AND METHODS

### Patients

Ninety-six consecutive patients with histologically proven primary gastric carcinoma who underwent surgery between January 1992 and December 1992 at the First Department of Surgery, Osaka City University Hospital, were studied. Peripheral blood samples were obtained from each patient upon admission to the hospital and, after centrifugation, the serum samples were stored at – 20°C until assayed. Serum samples from 100 healthy individuals were used as controls. There were no statistically significant differences in age and sex between patients and healthy controls; mean age ± SD, 58.2 ± 11.3 years vs 59.9 ± 10.9 years ($P = 0.530$); male–female, 67:29 vs. 75:25 ($P = 0.389$). Patients who had multiple cancers or any autoimmune disease were excluded from this study. No patient received neoadjuvant chemotherapy or irradiation. The patients with stage II to IV disease were given 5-fluorouracil orally after operation. All the pathological diagnoses and classifications were made according to the Japanese Classification of Gastric Carcinoma (Japanese Research Society for Gastric Cancer, 1995). All surviving patients were followed for more than 50 months. The survival period was defined as the interval from when the serum sample was obtained until 28 February 1997 for all living patients or until the day of death.

### Assay

The sera were assayed for sIL-2R with an enzyme-linked immunosorbent assay using Cellfree Interleukin 2 Receptor Kits (Yamanouchi, Tokyo, Japan) according to the manufacturer's instructions. In brief, a serum sample was added on a bead coated with a monoclonal antibody which relates to one epitope of sIL-2R. After incubation for 2 h, the sIL-2R fixed on the bead was reacted with horseradish peroxidase-conjugated monoclonal antibody, which binds a second epitope of sIL-2R. Following the sandwich assay, the colour reaction was terminated by addition of 2 N sulphuric acid and the absorbance was measured at 492 nm. Our reference value for sIL-2R was 639 U $ml^{-1}$ (the mean of 100 healthy controls plus one standard deviation). Serum carcinoembryonic antigen (CEA) levels and carbohydrate antigen 19-9 (CA 19–9) levels were measured by counting immunoassay using a commercially available CEA kit (Ranream CEA; TOA Medical Electronics, Kobe, Japan) and a CA 19-9 kit (Ranream CA 19-9; Toray–Fuji Bionics, Tokyo, Japan). The cut-off values of CEA and CA 19-9 levels recommended by the manufacturer were 6.5 ng $ml^{-1}$ and 37 U $ml^{-1}$ respectively. The white blood cell, lymphocyte and monocyte numbers were counted by a radio frequency/direct current detection method using an automated haematology analyser (SE-9000, TOA Medical Electronics, Kobe, Japan).

### Statistical analysis

The non-parametric Mann–Whitney *U*-test was used for the comparison of data between the two groups. The parametric Student's *t*-test was used for the comparison of age between the two groups. The survival data were estimated by the Kaplan–Meier method and examined by the log-rank test. The Cox proportional hazards model was employed for the multivariate analysis of survival. The chi-square test was used to compare the prevalence or distribution of two variables. The correlation between the sIL-2R level and tumour marker or blood cell number was assessed by linear regression using the least-squares method. A *P*-value of < 0.05 was considered statistically significant.

**Figure 1** The distribution of the serum soluble interleukin-2 receptor (sIL-2R) level according to stage of disease (I–IV), as defined in the Japanese Classification of Gastric Carcinoma, and healthy control subjects (H). The numbers in parentheses refer to the number of cases examined. There were significant differences between the serum sIL-2R levels of those with stage III/IV disease and those with stage I/II disease or the healthy control subjects by Mann–Whitney *U*-test ($P = 0.0002$ or $P = 0.0005$ respectively). The horizontal line represents the median value

### RESULTS

### Serum sIL-2R level and clinicopathological features in gastric cancer

The serum sIL-2R level in the patients with primary gastric cancer was 502 ± 254 U $ml^{-1}$ (mean ± SD) with a median value of 443 U $ml^{-1}$ (range 173–1880 U $ml^{-1}$). There was no statistically significant difference between serum sIL-2R levels in all patients with gastric cancer and in healthy control subjects

**Table 1** Association between clinicopathological factors and the serum soluble interleukin-2 receptor level.

| Features | Number | sIL-2R (U $ml^{-1}$) | | *P*-value |
|---|---|---|---|---|
| | | Mean ± SD | Median (range) | |
| Histology | | | | |
| Differentiated type | 44 | 503 ± 206 | 466 (173–1380) | 0.386 |
| Undifferentiated type | 52 | 501 ± 290 | 438 (186–1880) | |
| Peritoneal metastasis | | | | |
| Negative | 88 | 476 ± 210 | 436 (173–1380) | 0.012 |
| Positive | 8 | 792 ± 468 | 616 (362–1880) | |
| Hepatic metastasis | | | | |
| Negative | 90 | 483 ± 237 | 438 (173–1880) | 0.022 |
| Positive | 6 | 782 ± 353 | 726 (362–1380) | |
| Serosal invasion | | | | |
| Negative | 67 | 443 ± 161 | 421 (186–929) | 0.007 |
| Positive | 29 | 639 ± 359 | 601 (173–1880) | |
| Lymph node metastasis | | | | |
| n0,n1 | 78 | 475 ± 186 | 422 (186–1040) | 0.010 |
| > n2 | 18 | 845 ± 600 | 586 (173–1880) | |
| Lymphatic invasion | | | | |
| 1y0,ly1 | 66 | 446 ± 162 | 416 (186–929) | 0.015 |
| > ly2 | 30 | 626 ± 358 | 579 (173–1880) | |
| Venous invasion | | | | |
| v0,v1 | 86 | 466 ± 180 | 436 (186–1040) | 0.011 |
| > v2 | 10 | 812 ± 502 | 720 (173–1880) | |

Differentiated type includes papillary adenocarcinoma and well-differentiated and moderately differentiated tubular adenocarcinoma. Undifferentiated type includes poorly differentiated adenocarcinoma, signet-ring cell carcinoma and mucinous carcinoma. The *P*-values were determined by the Mann–Whitney *U*-test.

© Cancer Research Campaign 1998

Figure 2 Probability of survival for all stage gastric cancer patients in relation to their serum sIL-2R levels. The cut-off value between the high and low serum sIL-2R concentration was defined as 639 U ml$^{-1}$, which represents the mean plus one standard deviation of the serum sIL-2R concentration of healthy control subjects. A statistically significant difference in survival rate was observed between the high and low sIL-2R groups

Figure 3 Probability of survival for stage III/IV gastric cancer patients in relation to their serum sIL-2R levels. The cut-off value between the high and low serum sIL-2R concentration was defined as 639 U ml$^{-1}$, which represents the mean plus one standard deviation of the serum sIL-2R concentration of healthy control subjects. A statistically significant difference in survival rate was observed between the high and low sIL-2R groups

(mean ± SD 509 ± 130 U ml$^{-1}$, median 442 U ml$^{-1}$ (range 277–1220 U ml$^{-1}$). However, the median sIL-2R level was significantly higher in the stage III/IV gastric cancer patients (mean ± SD, 688 ± 356 U ml$^{-1}$; median 601 U ml$^{-1}$) than in those with stage I/II disease (mean ± SD 437 ± 164 U ml$^{-1}$, median 411 U ml$^{-1}$) or the healthy control subjects (Figure 1). There was no significant difference between the serum sIL-2R levels of those with differentiated cancers (well-differentiated and moderately differentiated tubular adenocarcinoma and papillary adenocarcinoma) and those with undifferentiated cancers (poorly differentiated adenocarcinoma, signet-ring cell carcinoma and mucinous carcinoma). However, there were significant differences in the serum sIL-2R levels between those with and without serosal invasion, peritoneal metastases and hepatic metastases. The serum sIL-2R level varied with the extent of lymph node metastases as well as lymphatic and venous invasion (Table 1).

### Serum sIL-2R level and prognosis in gastric cancer

When all patients with primary gastric cancer were divided into high and low groups using a cut-off serum sIL-2R concentration of 639 U ml$^{-1}$, the high sIL-2R group consisted of 19 patients and the low sIL-2R group consisted of 77 patients. The prognoses of the two groups were significantly different (Figure 2). In those with stage I/II disease, the prognoses of the high and low sIL-2R groups were similar because only two patients in both groups died from a recurrence of their disease. Conversely, there was a significant difference in the prognoses of the two sIL-2R groups with stage III/IV disease (Figure 3).

Nonetheless, the following clinicopathological features and treatments for the two groups were not significantly different by the chi-square test: stage III vs. IV, $P = 0.23$; differentiated type vs. undifferentiated type, $P = 0.09$; peritoneal invasion negative vs. positive, $P = 0.91$; hepatic metastasis negative vs. positive, $P = 0.41$; serosal invasion negative vs. positive, $P = 0.10$; lymph node metastasis n0, n1 vs. n2–n4, $P = 0.91$; lymphatic invasion ly0,ly1 vs. ly2,ly3, $P = 0.06$; venous invasion v0,v1 vs. v2,v3, $P = 0.51$; curability of gastric resection A (no residual tumours with high probability of cure) vs. B (no residual tumours but not evaluated as 'Curability A') vs. C (definite residual tumours), $P = 0.18$.

### Multivariate analysis for influence of the serum sIL-2R level on survival

Peritoneal, hepatic and lymph node metastases, depth of invasion, lymphatic and venous invasion, serum sIL-2R, CEA and CA 19-9 level, and peripheral blood mononuclear cells (PBMCs) number were analysed for all stage I–IV patients by the Cox proportional hazards model. A high serum level sIL-2R was an independent and strong factor which correlated with the prognosis of patients with primary gastric cancer (Table 2).

### Correlation of serum sIL-2R level with CEA or CA 19-9

The sensitivity (the number of positive patients with gastric cancer/the total number of patients with gastric cancer) of the serum CEA and CA 19-9 concentrations in our series were 31.3% and 6.3% respectively. The correlation coefficient values of the sIL-2R level with the CEA and CA 19-9 levels were 0.22 ($P = 0.03$) and 0.09 ($P = 0.37$) respectively.

### Correlation between the serum sIL-2R level and peripheral blood monocytes

There was a correlation between the serum sIL-2R level and the number of PBMCs ($r = 0.54$, $P < 0.0001$) (Figure 4). However, there was a weak or no correlation between the serum sIL-2R level and the number of white blood cells ($r = 0.20$, $P = 0.045$) or lymphocytes ($r = 0.0004$, $P = 0.997$).

### DISCUSSION

Although there have been many studies on the serum sIL-2R level in lung (Buccheri et al, 1991; Poulakis et al, 1991) and ovarian cancer patients (Barton et al, 1993, 1994; Gadducci et al, 1994), few studies have been reported on this cytokine receptor in the sera

© Cancer Research Campaign 1998

**Figure 4** The linear regression analysis between the serum sIL-2R titre and the number of PBMCs. The two values were correlated of gastric cancer patients. Lissoni et al (1990) found that metastatic gastric cancer patients had significantly higher serum sIL-2R levels than those with locally limited disease, and no significant difference was observed when the patients were grouped according to tumour histotype. Wang et al (1994) studied the sera from 45 gastric cancer patients and concluded that the mean sIL-2R level in gastric cancer patients was significantly higher than that in healthy controls, and that patients with metastatic disease had higher sIL-2R levels than those without metastatic disease. Murakami et al (1994*b*) analysed the serum sIL-2R levels of 40 patients with gastric cancer prior to surgery and found that the status of lymph node metastasis alone significantly influenced the serum sIL-2R level. We performed our study using more subjects and obtained the following results: patients with stage III/IV disease have elevated sIL-2R levels compared with those with stage I/II disease and with healthy individuals. There was no association between the histological type and the serum sIL-2R level. There were significant differences in the serum sIL-2R levels between the presence and the absence of peritoneal metastasis, hepatic metastasis, serosal invasion, lymph node metastasis, lymphatic invasion and venous invasion.

The mechanism responsible for the increase in the serum sIL-2R level in patients with advanced solid tumours remains to be elucidated. The serum sIL-2R level is thought to herald a surge of activated T cells (Rubin et al, 1985). If so, the increase in the sIL-2R level may be the result of immune response activation. In contrast, sIL-2R has been postulated to negatively modulate the host immune response (Rubin and Nelson, 1990). When the sIL-2R concentration increases in the serum, it competes with the cell-surface IL-2 receptor for binding to IL-2 and it may reduce the availability of IL-2 for IL-2-dependent immune responses (Rubin et al, 1985). The possible down-regulatory role of sIL-2R on T-cell function has not been confirmed and it has been reported that even high concentrations of sIL-2R are unable to block IL-2-induced T-cell proliferation in vitro (Pizzolo et al, 1992). Although there have been controversial reports, the competition between sIL-2R and the cell-surface IL-2 receptor may help to promote the growth of the primary tumour and any metastases; therefore, the serum sIL-2R level may correlate with a patient's prognosis. Buccheri et al (1991) reported that lung cancer patients with elevated serum sIL-2R levels (> 700 U ml$^{-1}$, the 93rd percentile value of healthy subjects) had a worse prognosis than those who did not. From their analysis of 85 patients with head and neck squamous cell carcinoma, Tartour et al (1997) suggested that the serum sIL-2R level at the time of diagnosis can serve as an independent prognostic indicator for the risk of locoregional recurrence and survival. (Tartour et al, 1997). Our results in gastric cancer patients were consistent with these previous reports. Patients with a high sIL-2R level (> 639 U ml$^{-1}$) had a significantly poorer prognosis than those who did not. Furthermore, an elevated serum sIL-2R concentration was a strong and independent predictor by multivariate survival analysis for the prognosis of patients with gastric cancer.

CEA and CA 19-9 are two of the most useful tumour markers for the diagnosis and monitoring of patients with gastric cancer. These tumour-associated antigens are shed from the tumour cell into the blood (Gold and Freedman, 1965; Koprowski et al, 1981). Similarly, sIL-2R was initially postulated to be released from activated T cells during a host immune response (Rubin et al, 1985). It has been reported that natural killer (NK) cells, activated B cells, PBMCs and eosinophils also express the p55 receptor (sIL-2R) (Waldmann et al, 1984; Holter et al, 1987; Rand et al, 1991).

Recently, it has been reported that various carcinoma cell lines express the α- and β-chains of IL-2R (Yasumura et al, 1994). In

**Table 2** Multivariate analysis of independent prognostic indicators in gastric cancer patients by the Cox proportional hazards model

| Variable | Coefficient | Standard error | (*P*-value) | 95% CI | Hazard ratio |
|---|---|---|---|---|---|
| Peritoneal metastasis | 0.379 | 0.477 | 0.4266 | 0.574–3.723 | 1.461 |
| Hepatic metastasis | 1.375 | 0.514 | 0.0074 | 1.445–10.828 | 3.956 |
| Depth of invasion | 0.256 | 0.356 | 0.4720 | 0.643–2.596 | 1.292 |
| Lymph node metastasis | 1.657 | 0.425 | < 0.0001 | 2.280–12.068 | 5.246 |
| Lymphatic invasion | 1.056 | 0.709 | 0.1365 | 0.716–11.534 | 2.874 |
| Venous invasion | – 1.747 | 0.638 | 0.0062 | 0.050–0.609 | 0.174 |
| Serum sIL-2R level (> 639 U ml$^{-1}$) | 2.342 | 0.841 | 0.0053 | 2.002–54.028 | 10.401 |
| Serum CEA level (> 6.5 ng ml$^{-1}$) | 0.322 | 0.835 | 0.6993 | 0.269–7.091 | 1.381 |
| Serum CA 19–9 level (> 37 U ml$^{-1}$) | 1.920 | 0.960 | 0.0456 | 1.038–44.784 | 6.818 |
| PBMC number (> 562 mm$^{-3}$) | 0.102 | 0.979 | 0.9169 | 0.163–7.546 | 1.108 |

CEA, carcinoembryonic antigen; CA 19-9, carbohydrate antigen 19-9; PBMC, peripheral blood mononuclear cell. The cut-off values were the means plus one standard deviation of healthy controls for sIL-2R and PBMCs, and the levels recommended by the manufacturers of the assay kits for CEA and CA 19-9.

© Cancer Research Campaign 1998

our study, the serum levels of sIL-2R were elevated according to the extent of the gastric tumour, raising the possibility that sIL-2R was produced by the tumour cells. However, the release of sIL-2R into the circulation from the carcinoma itself has not been firmly proven. Perhaps because of these differences in release, there was no, or only a weak, correlation between the serum sIL-2R level and the serum CA 19-9 or CEA concentration in those patients with gastric cancer. Thus, the serum sIL-2R concentration can be used as an alternative tumour marker in those with gastric cancer.

We recognized an association between the serum sIL-2R concentration and the PBMCs. The correlation can be explained in at least two ways. The first is that the increased PBMCs themselves release high levels of sIL-2R in the serum, while the second is that the increased PBMCs stimulate a lot of T cells, which shed much sIL-2R (Rubin et al, 1985). In any event, the PBMCs tended to be higher in patients with a high serum sIL-2R level. In contrast, the number of lymphocytes or white blood cells did not correlate with or correlated weakly with the serum sIL-2R concentration because these cell counts may not be affected by activated T cells or other immunoregulatory cells which release sIL-2R.

In conclusion, serum sIL-2R may be an independent prognostic indicator for patients with gastric cancer, especially those with advanced-stage disease. Further studies must address the question as to whether carcinoma cells themselves can release sIL-2R into the serum.

### REFERENCES

Ambrosetti A, Nadali G, Vinante F, Carlini S, Veneri D, Todeschini G, Morosato L, de Sabata D, Chilosi M, Maggi E, Parronchi P, Romagnani S, Semenzato G, Perona G and Pizzolo G (1993) Serum levels of soluble interleukin-2 receptor in Hodgkin's disease. Relationship with clinical stage, tumor burden, and treatment outcome. *Cancer* **72**: 201–206

Barton DP, Blanchard DK, Michelini Norris B, Nicosia SV, Cavanagh D and Djeu JY (1993) High serum and ascitic soluble interleukin-2 receptor alpha levels in advanced epithelial ovarian cancer. *Blood* **81**: 424–429

Barton DP, Blanchard DK, Wells AF, Nicosia SV, Roberts WS, Cavanagh D and Djeu JY (1994) Expression of interleukin-2 receptor alpha (IL-2R alpha) mRNA and protein in advanced epithelial ovarian cancer. *Anticancer Res* **14**: 761–772

Berghella AM, Pellegrini P, Piancatelli D, Maccarone D, Del Beato T, Giubilei D, Pomidori A, Adorno D and Casciani CU (1994) Progression mechanisms in colon cancer: soluble interleukin-2 (IL-2) receptor, IL-2 plus anti-CD3 proliferative response and tumour stage correlations. *Cancer Immunol Immunother* **38**: 160–166

Brown AE, Rieder KT and Wehster HK (1989) Prolonged elevation of soluble interleukin-2 receptors in tuberculosis. *Am Rev Respir Dis* **139**: 1036–1038

Buccheri G, Marino P, Preatoni A, Ferrigno D and Moroni GA (1991) Soluble interleukin 2 receptor in lung cancer. An indirect marker of tumor activity? *Chest* **99**: 1433–1437

Gadducci A, Ferdeghini M, Malagnino G, Prontera C, Fanucchi A, Annicchiarico C, Bianchi R, Fioretti P and Facchini V (1994) Elevated serum levels of neopterin and soluble interleukin-2 receptor in patients with ovarian cancer. *Gynecol Oncol* **52**: 386–391

Gold P and Freedman SO (1965) Demonstration of tumor-specific antigens in human colonic carcinoma by immunological tolerance and adsorption techniques. *J Exp Med* **121**: 439–462

Holter W, Goldman CK, Casabo L, Nelson DL, Greene WC and Waldmann TA (1987) Expression of functional IL-2 receptors by lipopolysaccharide and interferon-γ human monocytes. *J Immunol* **138**: 2917–2922

Japanese Research Society for Gastric Cancer (1995) *Japanese Classification of Gastric Carcinoma*. First English Edition. Kanehara: Tokyo

Koprowski H, Herlyn M, Steplewski Z and Sears HF (1981) Specific antigen in serum of patients with colon carcinoma. *Science* **212**: 53–55

Lai KN, Ho S, Leung JC and Tsao SY (1991) Soluble interleukin-2 receptors in patients with nasopharyngeal carcinoma. *Cancer* **67**: 2180–2185

Lissoni P, Barni S, Rovelli F, Viviani S, Maestroni GJM, Conti A and Tancini G (1990) The biological significance of soluble interleukin-2 receptors in solid tumors. *Eur J Cancer* **26**: 33–36

Murakami S, Satomi A, Ishida K, Murai H and Okamura Y (1994*a*) Serum soluble interleukin-2 receptor in colorectal cancer. *Acta Oncol* **33**: 19–21

Murakami S, Satomi A, Ishida K, Murai H, Matsuki M and Hashimoto T (1994*b*) Serum-soluble interleukin-2 receptor concentrations in patients with gastric cancer. *Cancer* **74**: 2745–2748

O'Garra A (1989) Interleukins and the immune system. *Lancet* **1**: 943–947

Pizzolo G, Vincenzi C, Vinante F, Rigo A, Veneri D, Chilosi M, Dusi S, Poli G, Zambello R, Semenzato G and Berton G (1992) Highly concentrated urine-purified Tac peptide fails to inhibit IL-2-dependent cell proliferation *in vitro*. *Cell Immunol* **141**: 253–259

Poulakis N, Sarandakou A, Rizos D, Phocas I, Kontozoglou T and Polyzogopoulos D (1991) Soluble interleukin-2 receptors and other markers in primary lung cancer. *Cancer* **68**: 1045–1049

Rand TH, Silberstein DS, Kornfeld H and Weller PF (1991) Human eosinophils express functional interleukin 2 receptors. *J Clin Invest* **88**: 825–832

Rubin LA and Nelson DL (1990) The soluble interleukin-2 receptor: biology, function, and clinical application. *Ann Intern Med* **133**: 619–627

Rubin LA, Kurman CC, Fritz ME, Biddison WE, Boutin B, Yarchoan R and Nelson DL (1985) Soluble interleukin 2 receptors are released by activated human lymphoid cells *in vitro*. *J Immunol* **135**: 3172–3176

Rubin LA, Jay G and Nelson DL (1986) The released interleukin 2 receptor binds interleukin 2 efficiently. *J Immunol* **137**: 3841–3844

Sharma S, Saha K, Shinghal RN and Malik GB (1991) Serum soluble interleukin-2 (IL-2) receptor levels in women with breast carcinoma and its correlation with IL-2 receptor expression on blood lymphocytes and lymphocytic infiltration within the tumour. *Cancer Immunol Immunother* **33**: 198–202

Symons JA, Wood NC, Giovine FS and Duff GW (1988) Soluble IL-2 receptor in rheumatoid arthritis: correlation with disease activity, IL-1 and IL-2 inhibition. *J Immunol* **141**: 2612–2618

Takeshita T, Goto Y, Tada K, Nagata K, Asano H and Sugamura K (1989) Monoclonal antibody defining a molecule possibly identical to the p75 subunit of interleukin 2 receptor. *J Exp Med* **169**: 1323–1332

Takeshita T, Asao H, Ohtani K, Ishii N, Kumaki S, Tanaka N, Munakata H, Nakamura M and Sugamura K (1992) Cloning of the γ-chain of the human IL-2 receptor. *Science* **257**: 379–382

Taniguchi T and Minami Y (1993) The IL2/IL2 receptor system: a current overview. *Cell* **73**: 5–8

Tartour E, Deneux L, Mosseri V, Jaulerry C, Brunin F, Point D, Validire P, Dubray B, Fridman WH and Rodriguez J (1997) Soluble interleukin-2 receptor serum level as a predictor of locoregional control and survival for patients with head and neck carcinoma. *Cancer* **79**: 1401–1408

Uchiyama T, Hori T, Tsudo M, Wano Y, Umadome H, Tamori S, Yodoi J, Maeda M, Sawami H and Uchino H (1985) Interleukin-2 receptor (Tac Antigen) expressed on adult T cell leukemia cells. *J Clin Invest* **76**: 446–453

Waldmann TA, Goldmann CK, Robb RJ, Depper JM, Leonard WJ, Sharrow SO, Bongiovanni KF, Korsmeyer SJ and Greene WC (1984) Expression of interleukin 2 receptors on activated human B cells. *J Exp Med* **160**: 1450–1466

Wang YF, Wu XN, Zhou YH, Chen XF, Shen J and Wang HJ (1994) Clinical significance of elevated serum soluble interleukin-2 receptor in gastric cancer. *Chin Med J Engl* **107**: 254–256

Yamaguchi S, Onji M and Ohta Y (1988) Increased serum soluble interleukin 2 receptor levels in patients with viral liver diseases. *Hepato-Gastroenterol* **35**: 245–248

Yasumura S, Lin W, Weidmann E, Hebda P and Whiteside TL (1994) Expression of interleukin 2 receptors on human carcinoma cell lines and tumor growth inhibition by interleukin 2. *Int J Cancer* **59**: 225–234

© Cancer Research Campaign 1998